# freeIbis: an efficient basecaller with calibrated quality scores for Illumina sequencers

**DOI:** 10.1093/bioinformatics/btt117

**Published:** 2013-03-06

**Authors:** Gabriel Renaud, Martin Kircher, Udo Stenzel, Janet Kelso

**Affiliations:** Department of Evolutionary Genetics, Max Planck Institute for Evolutionary Anthropology, D-04103 Leipzig, Germany

## Abstract

**Motivation:** The conversion of the raw intensities obtained from next-generation sequencing platforms into nucleotide sequences with well-calibrated quality scores is a critical step in the generation of good sequence data. While recent model-based approaches can yield highly accurate calls, they require a substantial amount of processing time and/or computational resources. We previously introduced Ibis, a fast and accurate basecaller for the Illumina platform. We have continued active development of Ibis to take into account developments in the Illumina technology, as well as to make Ibis fully open source.

**Results:** We introduce here freeIbis, which offers significant improvements in sequence accuracy owing to the use of a novel multiclass support vector machine (SVM) algorithm. Sequence quality scores are now calibrated based on empirically observed scores, thus providing a high correlation to their respective error rates. These improvements result in downstream advantages including improved genotyping accuracy.

**Availability and implementation:** FreeIbis is freely available for use under the GPL (http://bioinf.eva.mpg.de/freeibis/). It requires a Python interpreter and a C++ compiler. Tailored versions of LIBOCAS and LIBLINEAR are distributed along with the package.

**Contact:** kelso@eva.mpg.de

**Supplementary information:** Supplementary data are available at *Bioinformatics* online.

## 1 INTRODUCTION

A crucial step in the Illumina sequencing pipeline is basecalling: the generation of individual nucleotide sequences and associated quality scores, which measure the probability of a sequencing error, from raw intensities. The default basecaller provided by Illumina, Bustard, develops a model from the raw intensities and uses it to perform basecalling.

Alternative basecallers aimed at achieving a better performance than Bustard have been proposed (Whiteford *et al.*, 2009). These basecallers can be divided into those that apply a modelling strategy like Bustard (naiveBayescall, Kao *et al.*, 2009 or see Das and Vikalo, 2012 for a faster implementation) and All your Base (AYB) (Massingham and Goldman, 2012) and those that rely on supervised learning approaches (Ibis, Kircher *et al.*, 2009) or intermediate approaches (Altacyclic, Erlich *et al.*, 2008).

We introduce an update to our basecaller Ibis. FreeIbis replaces the restricted license SVM library with LIBOCAS (Franc and Sonnenburg, 2009), which is released under the GNU Public License. Our results show that freeIbis outperforms the previous version of our software in terms of sequence accuracy. We measured how the decision score of the SVM corresponded to the observed error rate as measured by the number of mismatches for each predicted quality score of control reads to their respective genome. A function approximating this distribution is then used to assign quality scores for individual bases. The resulting scores show a high level of correlation between their observed error rate and the predicted one, thus obviating the need for quality score recalibration as a post-processing step (McKenna *et al.*, 2010). We compare the newest versions of freeIbis and Ibis against the default basecaller for two Genome Analyzer II (GA) runs, a HiSeq run and a MiSeq run. On a set of DNA sequences genotyped using both Sanger and Illumina sequencing technologies, freeIbis provides an improvement in genotype accuracy over the default Illumina basecaller.

## 2 METHODS

The performance and accuracy of a number of freely available SVM libraries for basecalling were evaluated on a control lane of 51 cycles from a ϕX174 reference strain (sequence provided by Illumina Inc.) sequenced on a GAII.

An examination of our training data, built using ϕX174 control sequences, revealed that numerous mislabelled training examples (i.e. intensities representing a certain base but labelled as another) were present and could be attributed to two types of artefacts: genuine sequence errors and divergent bases in the control genome population. To eliminate the effects caused by the latter, a masking procedure for these positions on the genome of the organism used as control was devised. Any training example from a position with a mismatch to a given nucleotide with >10% of its coverage was removed.

As the divergent bases on the ϕX174 were masked, we sought to measure whether the posterior probabilities of the SVM corresponded with the observed error rate. However, standard implementations of the SVM algorithm do not output posterior probabilities but decisions values for each hyperplane. We implemented a method to convert these values into actual base quality scores (see Supplementary Methods). Alignments were performed using BWA version 0.5.8 a (Li and Durbin, 2009) with default parameters.

## 3 RESULTS

We compared freeIbis with the masking disabled to the most recent version of Ibis on the aforementioned run containing 200 000 sequences from a ϕX174 control lane with a high thymine retention (Kircher *et al.*, 2009). The reads produced by both versions were aligned back to the ϕX174 genome, and the number of sequences mapped and average edit distance was computed. We observed that LIBOCAS outperforms the previous SVM library for both metrics.

Because the introduction of incorrectly labelled training examples could influence the quality of the SVM model, we sought to evaluate whether our masking procedure would have an effect on the number of mapped reads. The mapping statistics confirmed that masking divergent bases on the ϕX genome improves the final sequence accuracy (170 572 sequences mapped) compared with not masking any bases (170 220) or masking random bases (170 225).

**Fig. 1.**
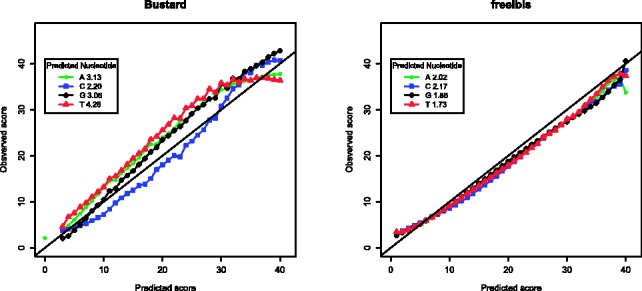
Plot of the predicted versus the observed base quality score for control reads. Ideally the base qualities should follow the diagonal line. The root mean square error (RMSE) shows that quality scores predicted using freeIbis have a greater correlation to their observed error rates

We tested freeIbis on a recent paired-end GAIIx run from mid-2011 from our own sequencing centre with 2 × 126 cycles and a single index of seven nucleotides. This multiplexed run had both human DNA as target, and ϕX174 as control and was basecalled using the previous version, Ibis, and the current one, freeIbis as well as naiveBayesCall (v. 0.3) and All your base (AYB, v2.08). We compared how each performed in terms of sequence accuracy, the number of sequences mapped and edit distance to the reference, as well as runtime (Table 1). We showed that freeIbis provides more high-quality base calls, leading to an increased number of reads being mapped to the reference with a lower edit distance than is the case for other basecallers. The predicted versus observed quality scores were plotted for Bustard and for freeIbis (Fig. 1). The sequences for the two GA runs used for comparison were produced using Bustard Off-Line Basecaller (OLB v.1.9.3). Our results show that freeIbis offers an improved accuracy and calibrated quality scores for these sequencing runs (including one on a HiSeq and another on a MiSeq) and outperforms Bustard on runs with unusually high error rates (see Supplementary Data).

**Table 1.** Accuracy for each basecaller on a Illumina GAIIx dataset (2 × 126 cycles with 366 135 257 clusters)

Basecaller	Training time	Calling time	Mapped (%)^a^	Edit distance
Bustard			583 348 201 (83.93%)	1.379
naiveBayesCall	591 h	658 h	578 957 145 (83.34%)	1.496
AYB	394 h		593 183 967 (85.52%)	1.076
Ibis	19.4 h	13.2 h	592 929 953 (85.31%)	1.167
freeIbis	21.3 h	12.2 h	594 095 219 (85.48%)	1.145

The human sequences were mapped to the hg19 version of the human genome. The number of mapped sequences and the average number of mismatches for those were tallied for each method. Time trials were conducted on a machine with 74 GB of RAM and using 8 of the 12 Intel Xeon cores running at 2.27 GHz. ^a^Percentage relative to sequences assigned to the read group of interest.

Using the genotype calls from the same sequencing data but using three different basecallers (Ibis, freeIbis and Bustard) to compare with calls from Sanger sequences, we determined that freeIbis offers improved genotyping accuracy (see Supplementary Data).

## 4 CONCLUSION

FreeIbis provides substantial improvements in sequence accuracy, quality score calibration and genotyping accuracy over Bustard, and is more computationally efficient than equally accurate model-based methods such as AYB.

## Supplementary Material

Supplementary Data

Supplementary Data
