# Supplementary material for: freeIbis: an efficient basecaller with calibrated quality scores for Illumina sequencers
Source: Bioinformatics. 2013 Mar 6;29(9):1208–9. doi: 10.1093/bioinformatics/btt117 (PMC3634191; doi:10.1093/bioinformatics/btt117)
Supplement: Supplementary Data [file supp_btt117_supplementary.pdf]

SUPPLEMENTARY METHODS

Quality Score Calibration

While the previous version of our basecaller sought to predict the quality based on empirically observed SVM decision scores and misclassification rates, the new version offers the possibility of calibrating SVM decision scores to observed errors using a logistic regression whose confidence probability score is, in turn, correlated to sequencing quality. This calibration is computed on every cycle and each nucleotide position within the sequence reads.

The library used for support vector machines produces, along with class assignment, decision score values associated for each label. One of the most standard methods for calibrating these values into actual posterior probabilities was proposed by Platt *et al.*, 1999 which proposes that this posterior probability can be modelled using a logistic function:

$$\frac{1}{1+e^{-z}}$$

As quality scores are computed on a PHRED scale defined as $-10 \cdot log_{10}(p_{error})$ and since the p_{error} is computed using the logistic function, plotting the input of the logistic function z against the observed error quality score would be expected to follow a somewhat linear relationship. However, we empirically determined that, despite this relationship being linear for the earlier scores, it reaches a quality score plateau induced by the background error rate of the procedure (see Figure 1). For high quality sequencing runs (e.g. a HiSeq with recent chemistry and normal cluster density) this plateau usually hovers around 40. In an attempt to model this distribution, a piecewise linear regression was used where one equation models the ascending quality scores due to higher SVM classification confidence while the other models the plateau which increases at a much lower rate than the former. This equation is subsequently used on reads to ascertain the quality score for each base. The resulting scores show a high correlation to their respective error rate. We found that even if a lane containing control reads is not used for this calibration, the high concordance between predicted and observed quality scores still holds.

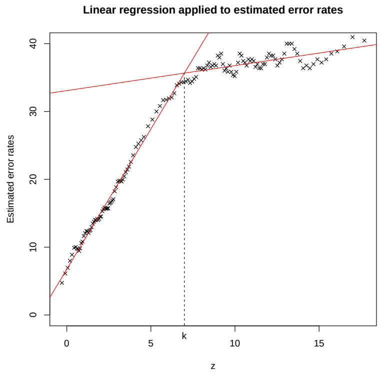

Fig. 1: Estimate of the error rate for control reads as a function of the input of the logistic function. A linear relationship would be expected between both variables, however, a plateau after reaching error rates of 40 is often seen thus the need to model this relationship using a piecewise linear regression. The observed error rate can be computed by sorting observations according to the value of the logistic function and computing the ratio of mismatches to observations for a given window. This process can be repeated using multiple windows to obtain estimates for various values of the logistic function. The value k represents boundary of the two subdomains of the piecewise linear function which are represented in red.

Comparing influence on genotype

To evaluate whether the new quality scores combined with the increased accuracy in basecalling would have any effect on the genotyping, we sought to compare the single nucleotide polymorphisms (SNPs) obtained from sequences basecalled using Ibis, freeIbis and the default basecaller provided by Illumina (Bustard) against genotype data from Sanger sequencing. We basecalled 3 different Illumina GAII runs from 2011 using the 3 aforementioned basecallers. The data was demultiplexed, stripped of sequencing adapters using an in-house sequencing pipeline (Kircher, 2012). For quality filtering, we used the overall likelihood of error from sequence quality scores and flagged the bottom 10% for each individual set as failing quality controls. From a panel of various individuals, we selected 10 individuals for comparison by the completeness of the genotyping obtained using the Sanger reads.

The data stemming from 49 genomic regions with a total length of 93kb (average: 1.9kb) from extant humans samples was mapped against the hg19 version of the human genome using BWA v.0.5.10 (Li and Durbin, 2009). The resulting data was genotyped using GATK v.1.3-14 McKenna *et al.*, 2010 (using option EMIT_ALL_SITES) after duplicate marking and removal using Picard v. 1.56 (http://picard.sourceforge.net) and indel realignment again using GATK. Given a general genotype quality cutoff value, the number of true positives, where Sanger and Illumina agreed, false positives (i.e. Illumina SNP but no Sanger), false negatives (SNP detected in Sanger but no alternative allele in Illumina) and true negatives were tabulated. Due to the presence of genuine SNPs which were not found in the Sanger data, only SNPs not found in dbSNP and with no clear sign of strand bias were tabulated as a false positive.

When comparing to the previous version of our software, the resulting genotyping accuracy (Table 3) presents less false positives at low quality but freeIbis produces more correct calls and better accuracy at higher genotype quality. This is due to the distribution of the quality scores (see Figure 8) between both basecallers as Ibis produces quality scores between the 20-30 range whereas freeIbis is able to confidently call bases at higher quality scores. At any genotype quality cutoff, freeIbis produces more correct calls and fewer erroneous ones than Bustard. Furthermore, the average genotype quality for all positions for freeIbis (58.98) is higher than Ibis' (58.77) or Bustard's (58.77).

On problematic data

To evaluated whether freeIbis would still have the robustness to improve the accuracy of a problematic dataset, we compared freeIbis to Bustard on a run with a high error sequenced on a Illumina GAIIx from our sequencing facilities at the Max Planck Institute for Evolutionary Anthropology (see Figure 2). The high error rate was due to an overloading of the flowcell thus making it arduous for the sequencer to delineate the different sequence clusters. We basecalled this run both with freeIbis and Bustard and compared the error rates for sequences identified as controls. Across lanes, the edit distance for reads basecalled with freeIbis had lower edit distance to their reference (see Table 2) and a greater percentage of sequences were mapped overall.

SUPPLEMENTARY DATA

Accuracy on Illumina sequencing data

Table 1. Sequence accuracy according to basecaller for all 4 platforms from different years

Basecaller	Mapped	(%)	Average edit distance
Genome Analyzer II 2009 (2x76 cycles)			
Bustard	101,487,701	(68.12%)	0.7757
naiveBayesCall	100,003,123	(67.12%)	0.8426
AYB	103,156,920	(69.23%)	0.6422
Ibis	101,093,708	(67.85%)	0.7702
freeIbis (logistic)	101,850,747	(68.36%)	0.7298
freeIbis (SVM)	102,091,337	(68.52%)	0.7205
HiSeq 2010 (1x100 cycles)			
Bustard	420,538,284	(83.71%)	0.8589
naiveBayesCall	423,616,381	(84,32%)	0.6962
AYB	431,426,132	(85.88%)	0.5148
Ibis	424,975,034	(84.59%)	0.7507
freeIbis (logistic)	424,592,468	(84.52%)	0.7826
freeIbis (SVM)	426,560,342	(84.91%)	0.7449
Genome Analyzer II 2011 (2x126+7 (index) cycles) [a] [b]			
Bustard	583,348,201	(83.93%)	1.3792
naiveBayesCall	578,957,145	(83.34%)	1.4960
AYB	593,183,967	(85.52%)	1.0755
Ibis	592,929,953	(85.31%)	1.1670
freeIbis (logistic)	593,312,238	(85.37%)	1.1640
freeIbis (SVM)	594,095,219	(85.48%)	1.1450
MiSeq (control sequences) 2012 (2x128+2x7 (indices) cycles)			
Bustard	273,642	(95.43%)	0.1844
Ibis	275,224	(95.41%)	0.1715
freeIbis (logistic)	282,569	(95.54%)	0.1665
freeIbis (SVM)	278,773	(95.24%)	0.1673

Every run, with the exception of the MiSeq one, used modern human DNA as sample. We therefore report the number of sequences that could be mapped back to the hg19 version of the human genome with the average edit distance to the reference. The percentage next to the total number of mapped sequences represent the fraction of the sequences pertaining to non-control lanes or, in the case of the 2 most recent runs, as a fraction of total number of sequences demultiplexed as belonging to the target sample. The average edit distance was computed using the NM field in the resulting BAM alignment. For every platform and various versions of the Illumina chemistry, the newest version of our software offers a significant improvement over Bustard in terms of sequence accuracy for having a greater number of mapped reads and a lower edit distance. For every run, the training was performed on the ϕX174 control sequences. For all but one of the runs, the reported number of aligned reads represents the number of human sequence reads aligning to the human genome reference. In the case of the MiSeq run, which used an ancient human DNA library with a low amount of endogenous human DNA, and which therefore had a small number of human sequences, the number of control reads aligning to the ϕX174 reference (provided by Illumina Inc.) are reported instead.

[a] This run was multiplexed thus the percentage of mapped reads represents the fraction out of the reads assigned to the desired read group that aligned to the human reference.

[b] The time reported in the main section for naiveBayesCall was calculated by multiplying, for all 8 lanes, the average time required for a single lane. Shorter training times can be obtained at the cost of sequence accuracy.

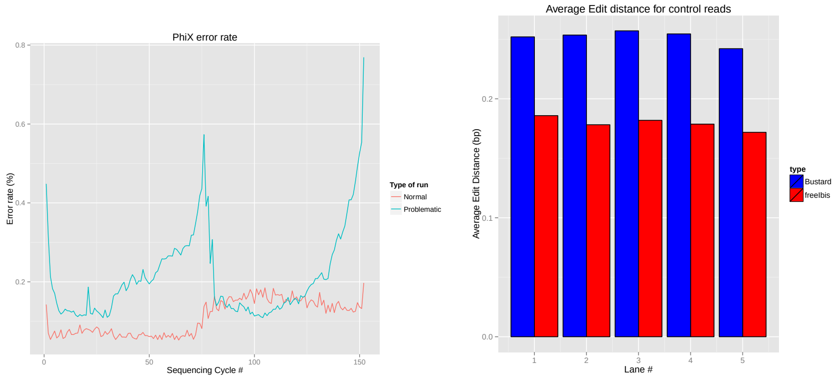

(a) (b)

Fig. 2: The error rate of control sequences for a problematic sequencing run (Illumina GAIIx 2x76bp) with an very high error rate (a) compared to different run (Illumina MiSeq 2x76bp) with a standard error rate. Although the error rate for control reads usually increases at the end due to increased phasing, it reaches for this particular run one error in 200 bases. The edit distance of these control reads to their reference genome (b) reveals that despite the increased error rate, freeIbis performs better than Bustard in terms of edit distance. For comparison purposes, the edit distance for the aforementioned MiSeq run with a standard error rate was 0.101632 thus revealing the problematic nature of this dataset.

Table 2. Percentage of sequences mapped for each basecaller

Basecaller	lane	number mapped	percentage mapped
Bustard	1	700,491	86.29%
	2	713,303	86.80%
	3	705,662	86.39%
	4	708,157	86.33%
	5	716,212	86.71%
freeIbis	1	711,741	87.93%
	2	724,318	88.41%
	3	717,236	88.21%
	4	719,325	88.10%
	5	727,228	88.59%

Percentage and number of mapped sequences identified as controls (for this multiplexed run, identified using the index sequences). Both in terms of number and percentages, sequences basecalled freeIbis have a greater tendency to map than the ones called with the default basecaller provided by the vendor.

Table 3. Genotype prediction accuracy according to basecaller at various genotype quality cutoffs

Basecaller:	Bustard				Ibis				freeIbis			
Genotype Quality	true pos.	false pos.	false neg.	true neg.	true pos.	false pos.	false neg.	true neg.	true pos.	false pos.	false neg.	true neg.
10	376	68	7	552,369	376	47	7	552,573	376	59	7	552,638
20	372	68	6	515,725	372	47	6	515,310	372	58	6	515,912
30	365	68	6	478,482	363	47	6	478,332	364	58	6	478,983
40	354	45	6	420,207	353	25	6	419,846	352	29	6	421,347
50	345	22	6	369,542	342	16	6	368,744	344	20	6	370,400
60	331	15	6	317,630	328	11	6	317,306	334	10	6	319,287
70	304	10	5	252,353	305	8	5	253,612	308	8	5	255,200
80	291	7	5	208,036	286	5	5	210,037	290	5	5	211,351
90	278	5	4	170,717	274	3	4	172,157	276	4	4	173,548

The accuracy of calling the genotype for 10 individuals which were genotyped using Sanger sequencing depending on the basecaller used for positive calls (pos.) and negative calls (.neg). At low genotype quality cutoffs, the previous version of our software minimizes the number of false positives due to the distribution of the quality scores. At higher genotype quality cutoff levels, Ibis fails to produce a large number of correctly predicted sites like freeIbis. However, at every genotype quality cutoffs, freeIbis offers more accurately predicted sites and fewer errors than the default basecaller.

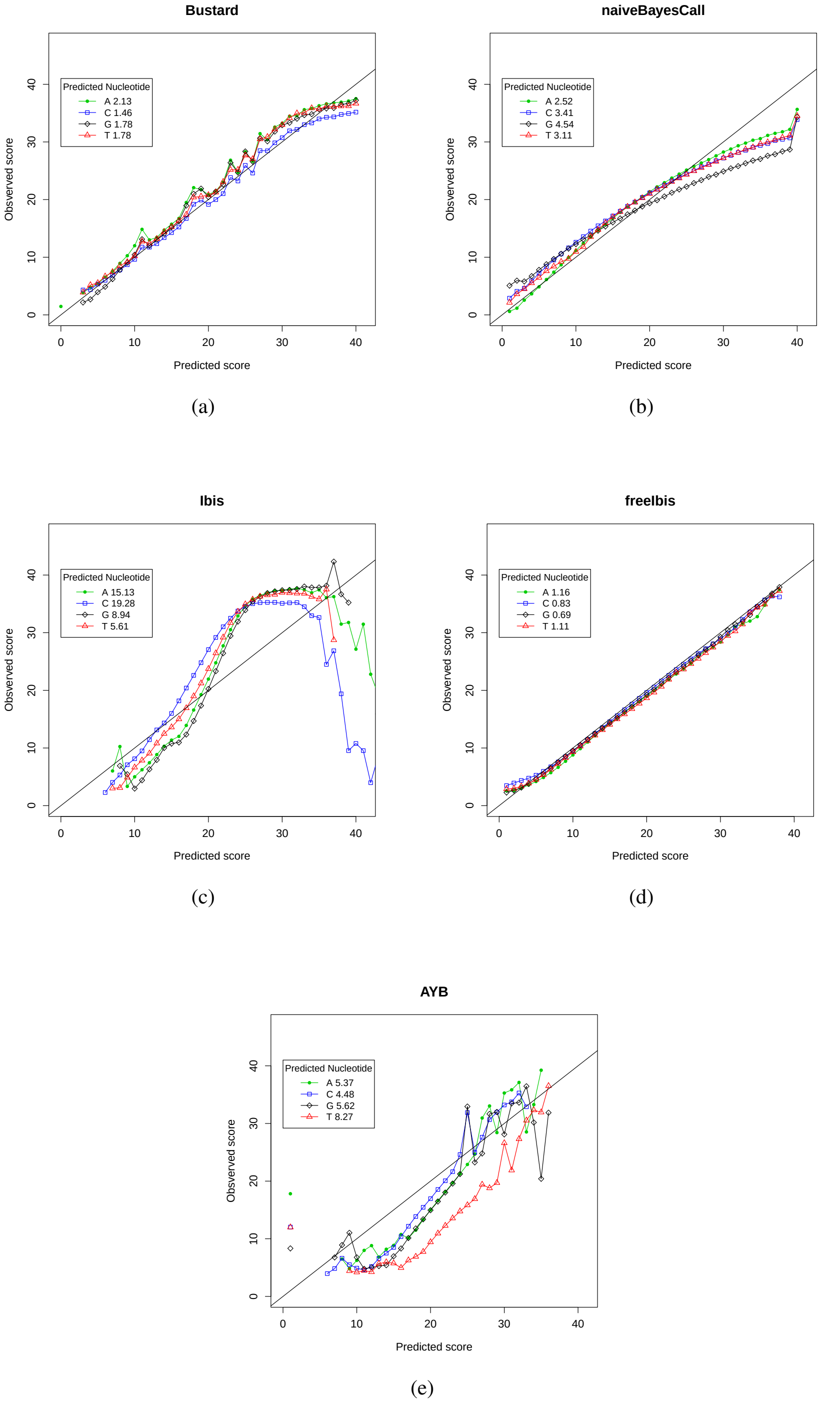

Fig. 3: The observed versus predicted quality scores for each nucleotide for the Genome Analyzer II (2009) run along with the RMSE. The graphs represent Bustard (a), naiveBayesCall (b), Ibis without calibration (c), freeIbis with calibration (d) and AYB (e). AYB provides a separate tool to recalibrate the quality scores based on observed quality scores based on clusters identified as controls. A downside of the freeIbis calibration method is, due to the shape of a the logarithm of the logistic function, an approximation using a linear function will underestimate data points around the origin and therefore, the actual error rate of bases with a low quality will be overstated (i.e. low quality bases have actually a higher observed quality score). This can be seen in (d) where low quality bases have an lower error rate than the predicted one and remain above the diagonal.

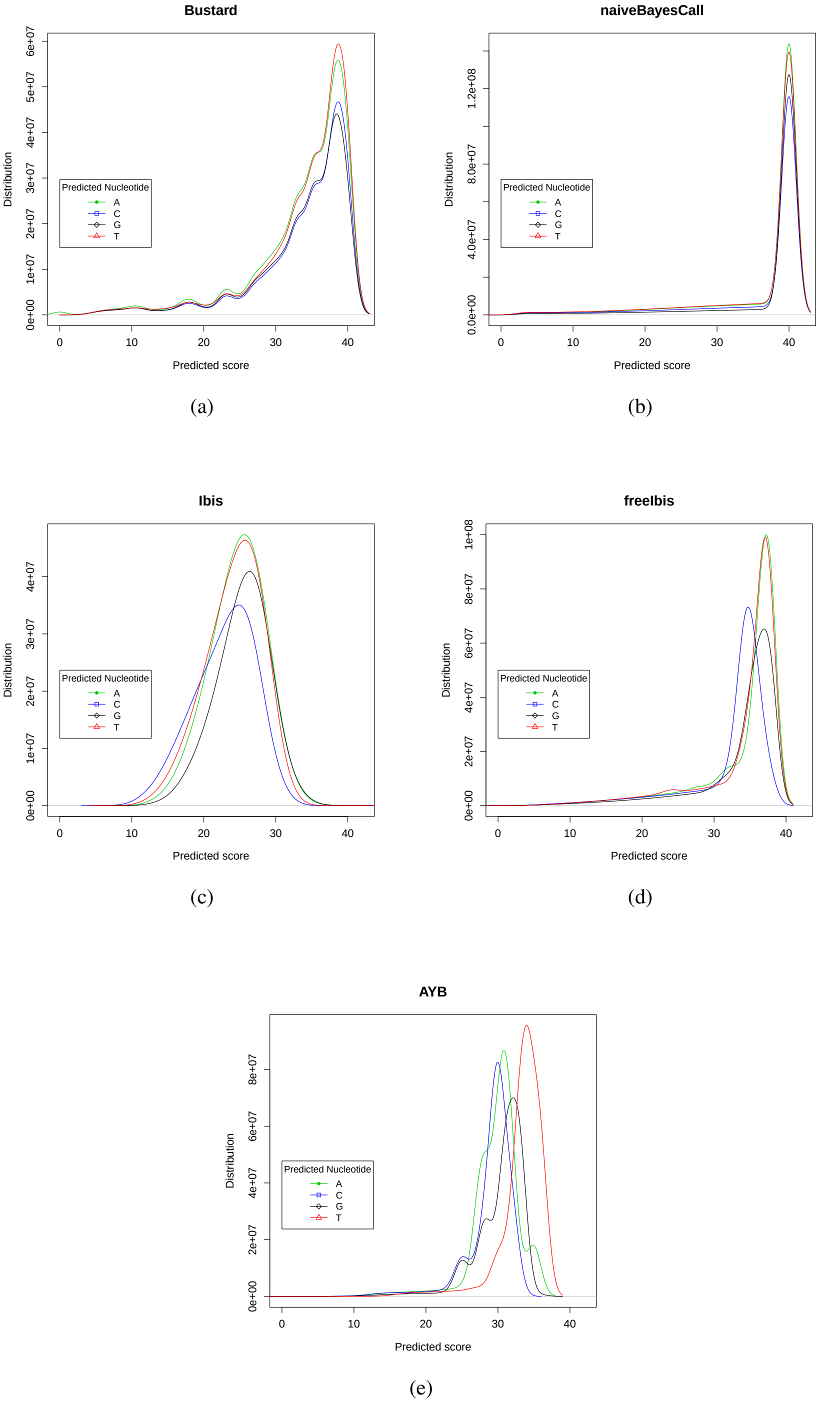

Fig. 4: The distribution of the quality scores for each nucleotide for the Genome Analyzer II (2009) run for Bustard (a), naiveBayesCall (b), Ibis without calibration (c), freeIbis with calibration (d) and AYB (e).

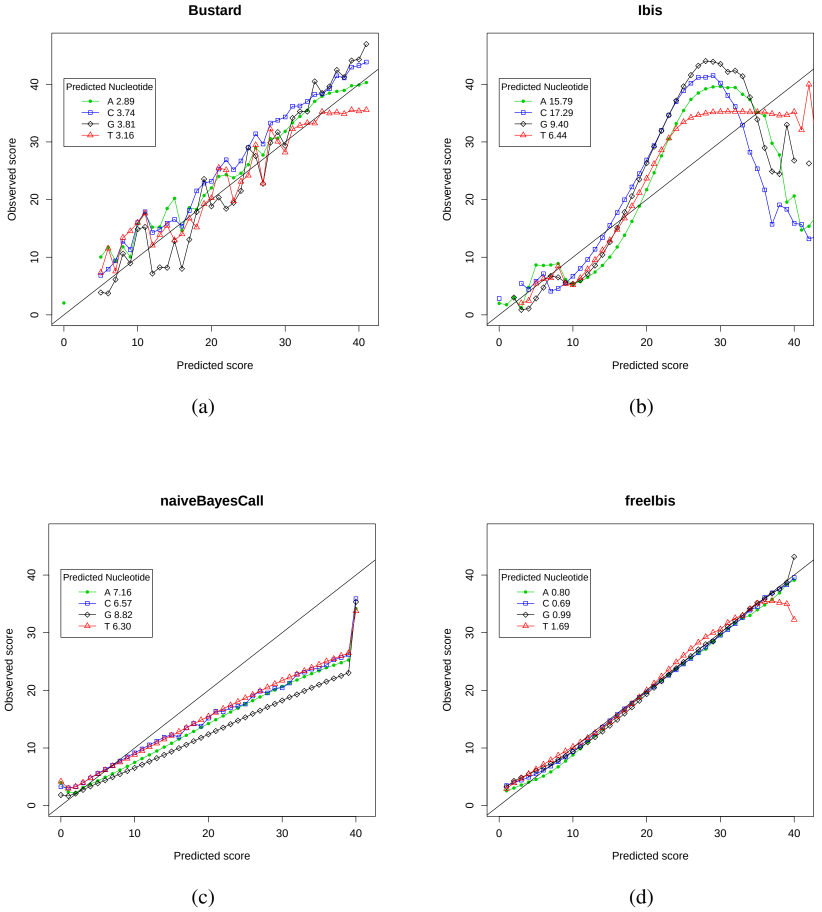

Fig. 5: The observed versus predicted quality scores for HiSeq (2010) for each basecaller namely Bustard (a), naiveBayesCall (c), Ibis without calibration (b) and freeIbis with calibration (d). AYB was unable to produce sequences for this control lane.

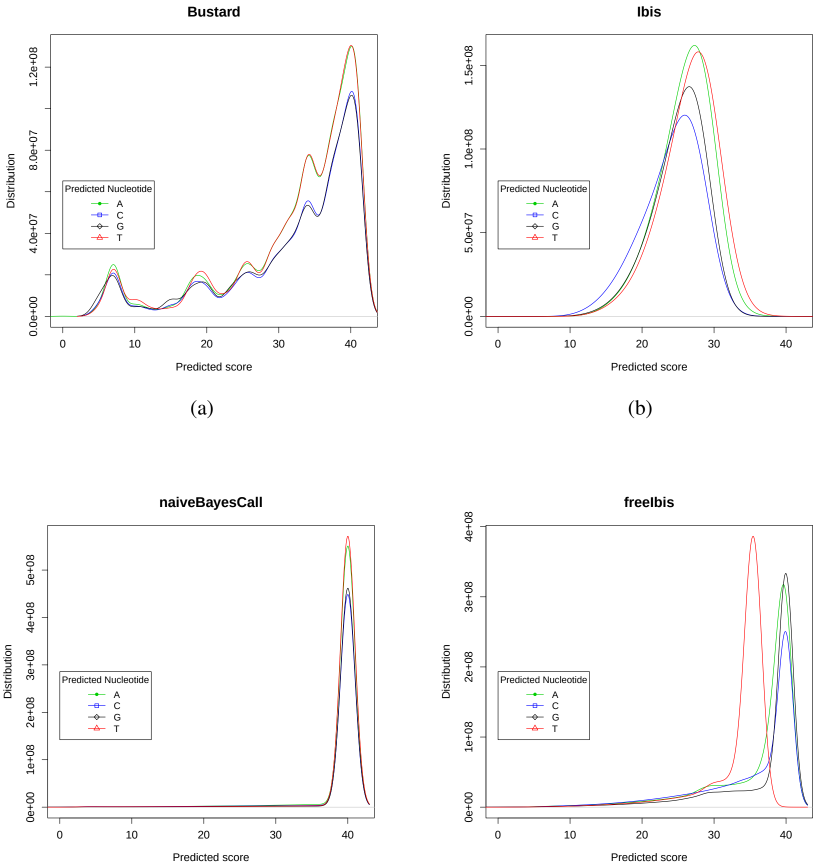

(a) (b)

(c) (d)

Fig. 6: The distribution of the predicted quality scores for HiSeq (2010) for Bustard (a), naiveBayesCall (c), Ibis without calibration (b) and freeIbis with calibration (d). The skewed distribution of the T nucleotide in the calibrated scores in freeIbis can be explained due to a higher error rate for this given nucleotide. AYB was unable to produce sequences for this control lane.

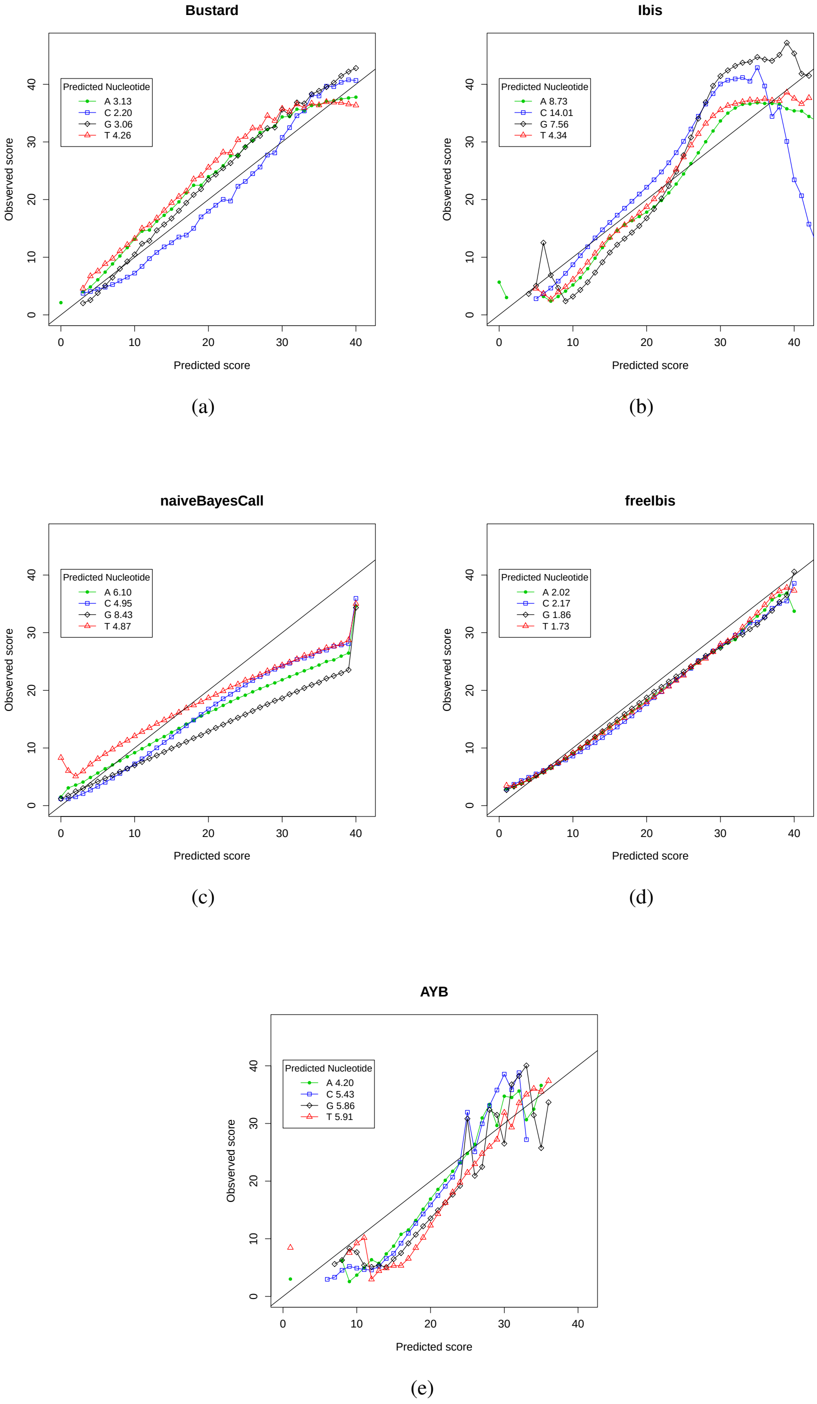

Fig. 7: The observed versus predicted quality scores plots for Genome Analyzer II (2011) for Bustard (a), naiveBayesCall (c), Ibis without calibration (b), freeIbis with calibration (d) and AYB (e).

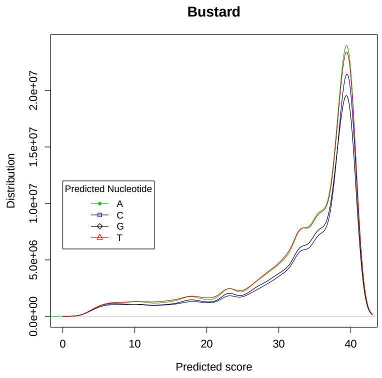

(a)

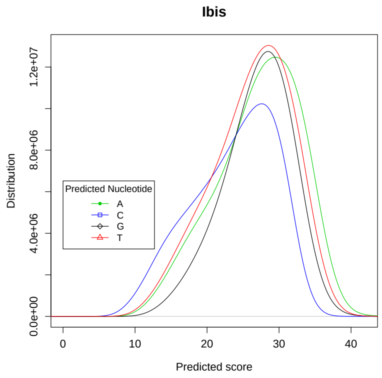

(b)

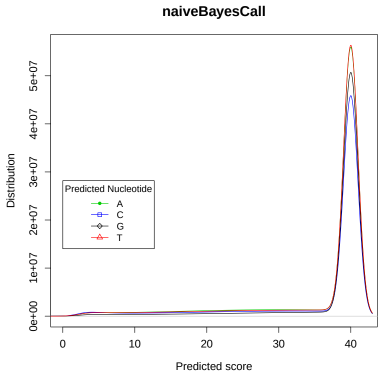

(c)

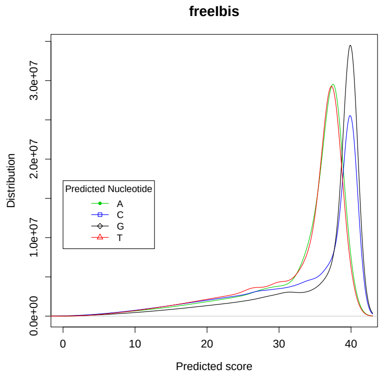

(d)

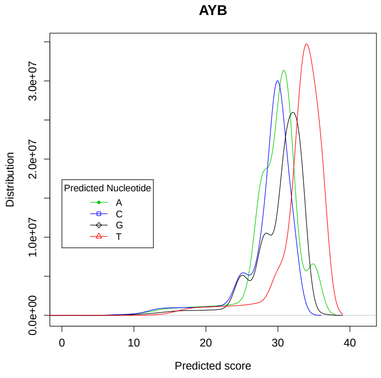

(e)

Fig. 8: The distribution of predicted quality scores for a sequencing run on the Genome Analyzer II (2011) platform (Bustard (a), naiveBayesCall (c), Ibis without calibration (b), freeIbis with calibration (d) and AYB (e)).

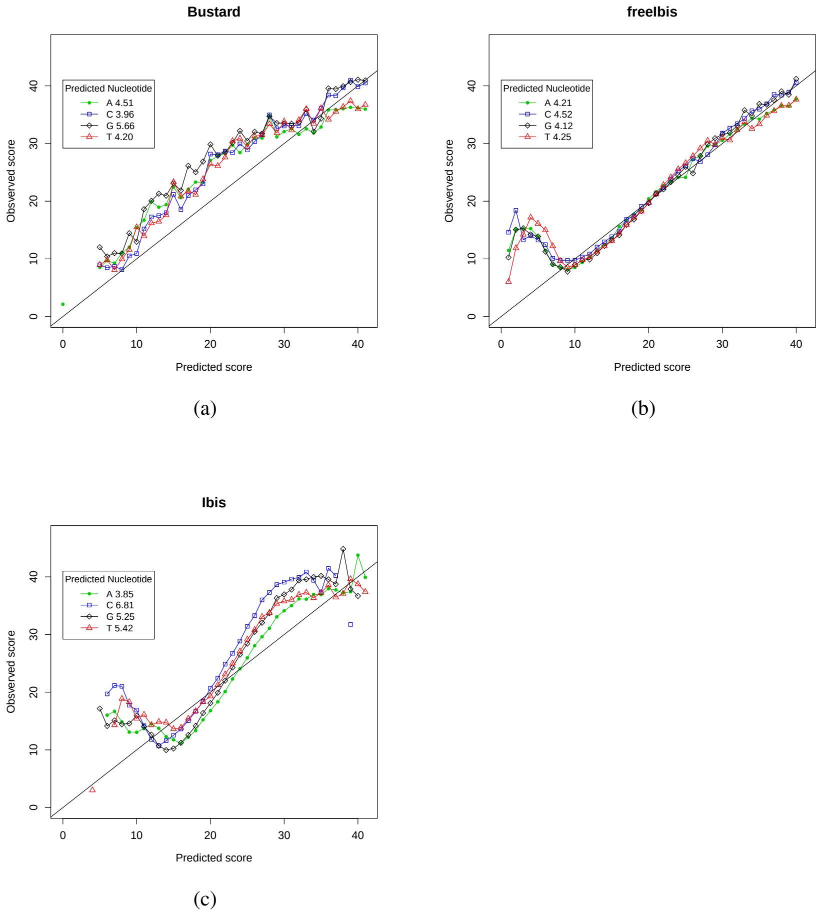

Fig. 9: Plots for the observed versus predicted quality scores for a sequencing run on the newest Illumina platform, the MiSeq (2012). The plots show the correlation for Bustard (a), Ibis without calibration (c) and freeIbis with calibration (b). Due to the paucity of control sequences needed to calibrate the quality scores, groupings of 5 consecutive cycles were used to measure the correlation between the SVM decision boundary distance and the observed error rate.

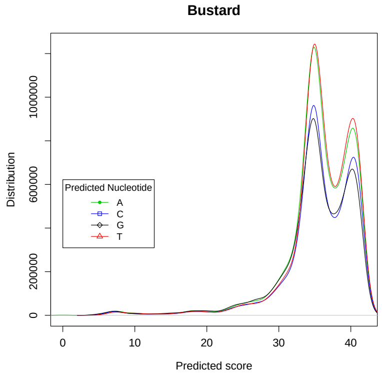

(a)

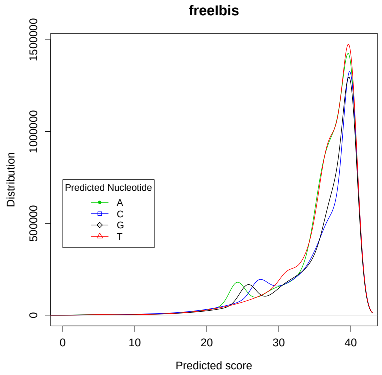

(b)

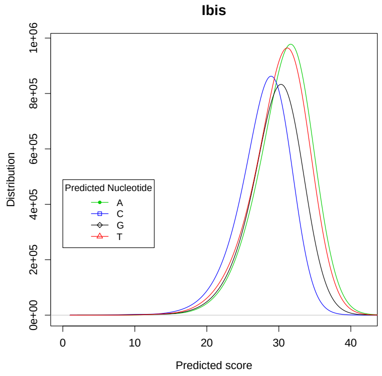

(c)

Fig. 10: Density plots of the predicted quality scores for the predicted quality scores on the MiSeq (2012) for various basecallers (Bustard (a), Ibis without calibration (c) and freeIbis with calibration (b)).

REFERENCES

Kircher, M. (2012). Analysis of high-throughput ancient DNA sequencing data. *Methods in molecular biology (Clifton, NJ)*, **840**, 197–228.

Li, H. and Durbin, R. (2009). Fast and accurate short read alignment with Burrows–Wheeler transform. *Bioinformatics*, **25**(14), 1754–1760.

McKenna, A., Hanna, M., Banks, E., Sivachenko, A., Cibulskis, K., Kernytsky, A., Garimella, K., Altshuler, D., Gabriel, S., Daly, M., *et al.* (2010). The genome analysis toolkit: A mapreduce framework for analyzing next-generation dna sequencing data. *Genome research*, **20**(9), 1297–1303.

Platt, J. *et al.* (1999). Probabilistic outputs for support vector machines and comparisons to regularized likelihood methods. *Advances in large margin classifiers*, **10**(3), 61–74.
