# Supplementary material for: freeIbis: an efficient basecaller with calibrated quality scores for Illumina sequencers
Source: Bioinformatics. 2013 Mar 6;29(9):1208–9. doi: 10.1093/bioinformatics/btt117 (PMC3634191; doi:10.1093/bioinformatics/btt117)
Supplement: Supplementary Data [file supp_29_9_1208_v2_index.html]

freeIbis: an efficient basecaller with calibrated quality scores for Illumina sequencers — freeIbis: an efficient basecaller with calibrated quality scores for Illumina sequencers — Supplementary Data 

# freeIbis: an efficient basecaller with calibrated quality scores for Illumina sequencers

## Supplementary Data

files

**Files in this Data Supplement:**

- Supplementary Data - pdf file
